# Supplementary material for: Effector innovation in genome-reduced phytoplasmas and other host-dependent mollicutes
Source: PLoS Genet. 2025 Nov 13;21(11):e1011946. doi: 10.1371/journal.pgen.1011946 (PMC12633904; doi:10.1371/journal.pgen.1011946)
Supplement: Attachment — Submitted filename: Response to reviewers_Mirkinetal.docx [file pgen.1011946.s032.docx]

We would like to thank the reviewers for taking their time to review this manuscript and for the considered feedback provided. We have addressed and provided a response on all points raised, as detailed below. Line numbers are given for alterations, which are also highlighted in the edited manuscript.

We have thanked the reviewers for their considerable efforts in the acknowledgements.

**Reviewer #1**: Markin et al. perform a systematic analysis to identify putative host-manipulating secreted effectors in the pangenome of phytoplasmas. The approach is rigorous and the results are informatively described by experts on the molecular biology of phytoplasma effectors. I think this is a high quality manuscript that will serve as a role model for similar systematic studies in other organisms.

Response: We thank the reviewer for their supportive comments.

##Minor comments / suggestions for improved clarity##

General—why “phytoplasma-associated” as part of the term for PhAMEs? Is this because “phytoplasma genomes” often contain host contigs due to their lack of culturability in axenic media?

Response: We agree with the reviewer that “phytoplasma-associated” may be confusing. However, we would like to keep the PhAME acronym, and changed it throughout the text as follows: Phytoplasma Effectors = PhAMEs.

L40 – grammar is awkward on “plant development, including witch’s broom and leaf-like flowers,”

Response: The grammar has been changed to “These effectors can suppress immunity and reprogram plant development, inducing alterations such as witch’s broom and leaf-like flowers, through ubiquitin-independent degradation of key transcription factors”.

General – Check the italics of taxonomic names like Wolbachia (L69) and Clostridium (L79), which are both genera. L352 candidatus. S. aureus in Figure 4H, and many instances in Figure 5. L623

Response: Taxonomic names have been italicized accordingly.

L103-L104 – It would be helpful for non-phytoplasma experts if you indicate whether the Potential Mobile Units (PMUs) have been demonstrated to be bona fide MGEs or not. If the evidence is “looks like a duck, quacks like a duck”, I suggest saying “lie within apparent mobile genetic elements”.

Response: Toruño et al show that the PMUs are mobile genetic elements, and this is cited at the first mention of PMUs.

T.Y. Toruño, M.S. Music, S. Simi, M. Nicolaisen, S.A. Hogenhout. Phytoplasma PMU1 exists as linear chromosomal and circular extrachromosomal elements and has enhanced expression in insect vectors compared with plant hosts. Mol Microbiol, 77 (2010), pp. 1406-1415.

L133 – Can you add a few words to clarify if “Membrane associated” means the proteins are associated with o embedded in the phytoplasma or the insect cell membranes?

Response: Text has been modified for clarity to “Moreover, phytoplasma trans-membrane proteins such as AMP (51,52), IMP (53) P38 (54) and PM19_00185 (55) play key roles in phytoplasma colonization of insect vectors.”.

L164 and in Table S1– can you indicate which markerset CheckM selected when the genomes were processed? In a different taxa, I have noticed that NCBI CheckM results are using a species-level marker set that seems to be biased (likely due to phylogenetic bias in the genomes used to define the set of single copy genes)

Response: Thank you for noticing this. In the previous version, we used the phytoplasma marker set. However, after reading the reviewer’s comment, we repeated the analysis using the Mollicute gene marker set after noting that the phytoplasma set uses 4 genomes, while the mollicute one has 119. Fig S1b and S1 Table have been modified accordingly. Used Marker set is clarified in materials and methods.

L166 – change “by all genomes” should be “by any of the genomes”; “by all genomes” means “core genes” in my opinion.

Response: The text was modified as suggested.

Figure S1b –I love your intuition to color the points based on this metric, but I found it a bit disappointing that so many genomes had “no data” for CheckM score. It’s a minor supplemental figure, so I don’t think you should necessarily re-run an analysis just to strengthen this figure.

Response: We agree and we re-made S1b Fig with the updated CheckM scores using the mollicute gene marker set. All genomes now have a reported CheckM score as indicated above.

L176 – I think you cited the wrong supplemental table. S5 Table seems to include the functional annotations. You might change “failed to identify domains” to “failed to identify functionally informative domains” because signal peptides count as a domain, they just don’t provide much information about function.

Response: Thank you for catching this. Tables are now cited correctly, and the text has been modified as suggested. Table 4 and 5 have been swapped and the index has been corrected.

L179 – is “(sC)” a typo? I think you started using “subC” as an abbreviation.

sC is the correct term.

Response: The abbreviation subC in S4 table has been changed to sC.

L180 and L187 – Table S5 does not include the subcluster IDs. I think you should cite Table S4 here. Since I noticed two of these easy-to-make mistakes, I encourage you to carefully check each figure and table reference to make sure readers will find the information.

Response: Thank you for catching this. Tables are now cited correctly as indicated above.

L224 “wondered of” should be “wondered if”. Google Docs’s spellcheck is good at catching this type of hard-to-notice typo.

Response: Typo has been corrected.

L224-L237 – No critique major here, but I wanted to let you know that I appreciated your careful self-critique of your approach.

Response: Thank you.

L475 – period is missing.

Response: Period was added.

L499 – This sentence says there were 2-24 scorpion-hp repeats, but Figure 4C indicates that many of the clusters had 0 of these repeats identified. I think the results sentence could be more clear.

Response: The sentence has been modified to more clearly express the number of repeats in PhAMEs showing repeats only refers to repeat-containing PhAMEs. The existence of PhAMEs within those clusters without repeats is indicated in Fig 4C and was stated in Lines 514-516 “Notably, other PhAMEs with β-hairpins, including those with membership to clusters C3 and C46, were not found to have Scorpion ꞵ-hp repeats (Fig. 4I-J), suggesting higher sequence divergence or structural convergence.” This now has been moved to Line 501 for increased clarity.

Additionally, C481 was included in F5C.

L625 – “acetylates”, not “acetylate”

Response: Typo has been corrected.

L809 – attB1 and attB2 should be italicized here. Also L819

Response: attB1 and attB2 have been italicized accordingly.

A curiosity that I had as a non-expert: I wonder if the “population bottlenecks” are a good thing for phytoplasmas. Those transmission events might allow the populations of these bacteria to keep more orderly genomes than the Hodgkinia found in long-generation cidadas. https://www.pnas.org/doi/10.1073/pnas.1421386112

Response: Yes, we like this thought. Not sure how orderly genomes apply to phytoplasmas as they appear to be highly dynamic as described here (e.g. Bai et al., 2006; Hogenhout and Seruga Music, 2010).

This was a very well-written manuscript. I want to thank you for pre-printing it so that I could ethically share it with my lab. It’s a role model for rigorous science and effective communication.

Response: Thank you for these nice comments. We are excited that you shared the manuscript with others – that what we hoped for by preprinting it in Biorxiv.

**Reviewer #2**: This work presents novel data and offers new insights about phytoplasma effectors. A great feature of this paper is that it addresses the whole effectorome of phytoplasmas, and thus is of interest for the whole phytoplasma research community (among others). A detailed structural basis for the activity of the already well-documented phytoplasma effectors SAP05, SAP54/PHYL1, SAP11 effectors is presented and stands as a complement to what was known already. New structural classes of yet uncharacterized effectors are unveiled. Authors bring evidence that "the overall effector repertoire of phytoplasmas appears largely unique".

The paper is well written, and provided data support the conclusions. Figures are rich and appealing.

Below is a listof several observations about discrepancies between text and supplemental data, or between text and figures, but none of them addresses the underlying approach of the work, or cast doubts on the conclusions drawn by the authors.

Response: We thank the reviewer for the supportive comments. We have addressed the concerns as per below.

A surprising observation is that supplemental tables S2 and S4 appear to contain duplicate entries, and the authors seem to be unaware of it. As a result, several protein counts may be overestimated (details below). The conclusions of the paper are still standing however.

Here are my comments, sorted by order of appearance in the manuscript.

And the related comment:

* p5 Section title. "Mining phytoplasma genome sequences identified 7162 PhAMEs". Table S4 lists the mentioned 7162 PhAMEs. However, analysis of table S4 showed that some entries are duplicated (for instance WP_213680437.1 is listed 20 times, and WP_004994795.1 19 times), likely due to the pooling of all phytoplasma proteins without removal of the duplicates. Therefore, the total number of PhAMEs as indicated in the title is overestimated (according to table S4, the total number of PhAMEs is rather 6362). Fortunately, this has no heavy consequence on the remaining of the analysis, thanks to the subsequent clustering step. However, the different counts indicated in the manuscript may be affected, as well as associated figures (Fig S2, etc. see also other comments below).

Response: We understand that this is confusing. However, ‘WP_213680437.1’ correspond to an entry code from RefSeq, which group proteins with identical sequences from different genomes under the same ID. In this example, the 20 ‘duplicates’ are derived from different phytoplasma genomes or from duplicated genes in the same genome. Therefore, we have kept the ‘duplicates’ as a true representation of the effector presence. The same is true for WP_004994795.1 and possibly other PhAMEs. Moreover, as the reviewer correctly observed, the ‘duplications’ were addressed as redundancy during the sub-clustering steps and so did not affect the downstream analyses. In summary, we think it is better to keep the ‘duplications’ in the tables and figures. Nonetheless, to better describe the diversity, we modified Fig. 1A, it’s corresponding legend and text to clearly state the number of distinct mature sequences. Additionally, we modified S4 table to include an additional column (Sequence-distinct_mature_PhAME_group) that assigns a unique number identifier to each sequence-distinct PhAME.

While including the above changes, we noticed minor inconsistencies. These were: (i) designations of ABC65342.1, QKX95545.1, WP_156956364.1, MBX4180509.1 to clusters C544, C555, C555 and, C556, respectively; and (ii) designations of WP_023161221.1, MBX4180509.1, QKX95545.1, WP_156956364.1, WP_122225569.1 to sub-clusters sC891, sC892, sC893, sC893 and sC894, respectively. These corrections raised the number of clusters from 553 to 556 and subclusters from 890 to 894. Text and figures (Fig 1B, S2 Fig, S3A Fig, S5B Fig, S5C Fig, S5D Fig) were corrected to reflect the minor changes in percentages.

* p5 line 20 "phytoplasma-associated molecular effectors". In their manuscript, the authors designate the putatively secreted proteins as well as demonstrated effectors by the term "PhAME", defined as "phytoplasma-associated molecular effectors". I am not really convinced that this new term was required, as these are not new concepts or new biological objects, and I wonder why the authors did not simply referred to them by "phytoplasma effectors" (assuming that the term effector can be applied even if secretion or biological role has not been demonstrated).

Response: As per response to a similar comment of reviewer 1, we changed the PhAME acronym throughout the text as follows: Phytoplasma Effectors = PhAMEs.

* p6 line 4 Title "PhAMEs group into 553 clusters". It seems that in provided table S4, accession ABC65342.1 has no defined cluster (leading to a count of 552 regular clusters). Maybe this cluster was inadvertently deleted in the table.

Response: Thank you for noticing this. See response above about ABC65342.1.

* p6 line 5 "Functional annotation…" Table S4 is cited but should be Table S5 according to supplemental tables numbering. The same confusion is present in several places in the manuscript and in the table of contents of supplementary tables, so I believe that somehow the tables S4 and S5 have been exchanged in the supplemental tables file provided by the authors. I invite the authors to check the references to tables S4 and S5 throughout the manuscript, and/or correct the supplemental table file.

Response: Tables are now cited correctly, and the text has been modified accordingly. Table 4 and 5 were swapped and the index was corrected accordingly.

* p6 line 10 "1518 representative PhAMEs were selected". However, according to table S4, this number is 1524 (5638 entries with "Not_evaluated_pipeline" in Average_pLDDT column out of 7162). Part of the discrepancies may be explained by entries not associated with any cluster (subcluster "#N/A")?

Response: Thank you for noticing these. The number was corrected to 1525 and Figure 1A. Additionally, Fig 1A now state the number of distinct predicted proteins. L181 now states that 1309 distinct representative PhAMEs were modelled.

* p6 line 17 "indicating that there is no obvious bias in the prediction quality of the secretomes". I am not sure whether this sentence refers to Fig S3A or S3B. I would suggest mentioning the figure referred to and reformulate the conclusion as it is not totally explicit.

Response: The reference is to Figure S3B. In Fig S3, Fig S1a and S4 table we now correctly assigned GCF_005774685.2 to 16Sr group IV and not XXI.

* p7 line 5 "Of the 7255 identified PhAMEs…" This number is inconsistent with the 7162 PhAMEs announced earlier in the text, and also is different from the number observed in supplemental data provided by the authors (see comment on table S2).

Response: Thank you. 7255 has been corrected to 7162.

* p7 line 7 Reference to table S5 is incorrect, should be table S4 (see above comment)

Response: Tables are now cited correctly, as indicated above.

* p7 line 16 "We next wondered of…" -> change "of" to "if"?

Response: Yes, indeed, typo was corrected.

* p8 line 15 "C3 and C36". C36 is not consistent with Supplemental fig S6C where C46 is mentioned instead.

Response: C36 has been modified to C46.

* p8 line 31 "The loop surface exhibits greater sequence variability…" text is associated with Fig S7B but should be Fig S7D?

Response: Yes, thanks. Text now refers to Fig. 7D.

* p9 line 1 "The loop surface structure of SAP05…" this title appears to lack some words ("… recognized by DNA-binding interface....?) and should be corrected (compare with line 11).

Response: We modified the title as suggested.

* p9 line 28 "preducted" should be changed in "predicted"

Response: Typo has been corrected.

* p10 section "Opposite facing surfaces of SAP54…". In this section, the authors use SAP54(OY-M)/PHYL1 in their structural models. However, in the next section ("Structural model validation…"), authors use SAP54(AYWB) for Y2H experiments. I guess that authors used the protein from strain OY-M, and not AYWB, for modelling because a crystal structure was available for OY-M (Iwabuchi et al. 2019). But this is not explained in the text. Moreover, a sentence (or reference) detailing how different the two proteins are could help to justify this choice.

Response: Yes, this is correct. The text now states that SAP54/PHYL1 from (OY-M) was used for the modelling and includes a comparison between the structure and sequence of the OY-M and AYWB proteins. Moreover, S12 Fig. shows conservation of the mutated residues between the two.

* p11 lines 20 and 21. Text refers to fig S10M, which does not exist, and should be S10J. Same with S10N (S10K).

Response: This has been corrected.

* p13 line 4 "S13M" should be replaced by S13I.

Response: Yes, this has been corrected.

* p14 line 9. "SAP11-like proteins are distributed across clusters C8, C85, C582", whereas Fig S16A indicates C8, C85, C445. Moreover, cluster size has to be corrected (according to provided table S4, cluster C8 contains 154 entries). I let the authors check all cluster sizes in the manuscript, including associated figures.

Response: The text has been modified from C582 to C445. Cluster C8 has 180 members as mentioned in the text.

* p14 line 26 "284 and 22 PhAMEs respectively". Due to the presence of duplicates in table S4, as already mentioned, correct number for cluster C3, according to table S4, should be 253. Text should be updated accordingly, but this may also have an impact on several associated figures of the manuscript.

Response: See response to the first comments of the reviewer.

* p15 line 10 "the scorpion beta-sheet domain has structural similarities to the dimerization domains of SdgB". Were there any other hits indicated by Foldseek when looking for structures similar to the scorpion domain?

Response: Yes, beyond the SdgB that is a glycosyltransferases from *Staphylococcus* *aureus* (as was mentioned in the text), the scorpion beta-sheet domain has also structural similarities to the dimerization/multimerization domain of other glycosyltransferases found in *Streptococcus* and other *Staphylococcus* species (Foldseek E-values of -15 to -5). We have changed the text to include this.

* p15 line 23 / Fig 5A "Our analysis identified shared folds between phytoplasma PhAMEs and proteins from other Mollicutes". Apart from interesting examples of proteins involved in interaction with the host highlighted by the authors, it is not precised whether the other PhAMEs structural homologs detected in Mollicutes are associated or not with a putative secretion signal. It is possible that some of these folds are associated with cytosolic proteins, in mycoplasmas/spiroplasmas/acholeplasmas. But it is the same for phytoplasmas, consistently with the genomic scenarios presented by the authors (fig 2J, 4G). That is why I would appreciate that authors provide (as supplemental data) a heat map similar to fig 5A, but with the 24 representative RePh phytoplasmas for the phylogenetic tree, and if possible with the proportion of proteins predicted to have a signal peptide. This would allow to compare phytoplasmas and remaining Mollicutes on the same grounds, and possibly add further evidence for the genomic evolution/origin of PhAME folds suggested by authors. Comparison of folds shared by effectors and cytosolic proteins within phytoplasmas might also shed a bit of light on the origin of some effectors.

Response: We agree with the reviewer. We added an extra 6 rows at the top of Fig. 5A. These new rows include the proteomes of 6 representative phytoplasmas (RePh). Moreover, we provide an additional supplemental figure (S17 Fig) that includes more clusters, including the ones unique to phytoplasmas. Additionally, we provide S12 table, which contains information about secretion and membrane localization of proteins listed in S17 Fig.

* p15 line 28 "PhAME clusters C122, C480, C605, C610 shared domains…". In fig 5A, clusters indicated to contain hIg-like folds are C122, C485, C480, C310.

Response: Yes, thanks, cluster numbers were corrected.

* p21 line 16 "cleavage site probability > 50" -> presumably 0.50

Response: >50 was corrected into ≥ 0.50

* p22 line 13 "database containing available from": some words are missing.

Response: Sentence was corrected.

* p22 line 32 "algorithm

Response: Not sure what the reviewer is referring to. Incomplete sentence?

* Fig1C-J Please confirm that used colors only indicate relative distance to N/C terminus.

Response: Figure legends have been modified to clarify ‘rainbow’ colouring (F1c-j, S6 Fig a-c, F3f, S9b Fig, S12 b, S13 b-g, S15 a, S16c, F4 i-j, F5 b-k, s18b fig (former S17b fig).

* Fig1B There is no arrow connected to SJP39 legend.

Response: Arrow added to indicate SJP39

* Fig2A – Legend. Replace "yellow" by "cyan"?

Response: Yellow has been replaced by cyan.

* Fig3D There is one dashed line without legend (PHYL1/SAP54OY-M).

Response: Legend was added.

* Fig3E and Methods. Two different media for yeast growth are indicated : "SC – LWHA + 10 mM 3AT" and "SC – LWH + 40 mM 3AT". Although this is not a problem, as the authors do not directly compare the two subfigures, and K106E shows the same behavior on both media, I was wondering why different media were used and if this choice was dictated by affinity differences between tested proteins. Moreover, the Methods section (p24) only mentions SD –LWH + 25/60/75 mM 3AT, thus is not consistent with media indicated in Fig3E.

Response: No, protein interactions are consistent, and we are not aware of affinity differences of the proteins depending on selective media used. The selective media were different, because the experiments were done at different times. Materials and methods were corrected to reflect the conditions used.

* Fig5A The size of black circles in the row of "Phytoplasma" is somewhat misleading compared to the white circles, because most clusters indicated below the heat map have only few members (sometimes only 1). Therefore, it would be more informative to have an idea of the size of the cluster/number of proteins involved, and would substantiate the sentence "the overall effector repertoire appeared largely unique" in discussion. Please see also my related suggestion where I propose to indicate PhAMEs as well as structural homologues not predicted to be secreted ("p15 line 23 / Fig 5A").

Response: This issue has been addressed by changing Fig. 5A as described above. There are no black circles any longer.

Fig5B-G The names of bacterial species should be written with italics. Also, some phytoplasmas have poor designation : "OY-M" and "AY-WB" indicate strains only. "Phytoplasma dorée" for sure refers to flavescence dorée phytoplasma, but is not a correct formulation. Also the accession number for VmpA (fig 5E) seems strange ("ostparc").

Response: We agree. We added *Ca.* Phytoplasma IDs to all names in Fig. 5.

* Fig5 legend. "M. malelucae" -> "M. melaleucae".

Response: Name has been corrected.

* Supplementary data. Submitted work relies on a large number of predicted structures currently not provided as supplemental data. I suggest that authors also consider releasing the predicted structures as supplemental data, if possible given the technical constraints. Note: I realized that authors already indicated in the submission form that they were willing to provide all of the predicted structures.

Response: Yes, we agree and all the predicted structures of the PhAMEs listed in S4 Table have been uploaded into Zenodo (doi: https://doi.org/10.5281/zenodo.17379298) as a dataset and is indicated in the new section ‘Data, Materials, and Software Availability’. The reference this Zenodo release has also been cited in the paper (Reference 137).

* S1 table. In the manuscript page 5, 1st paragraphe of "Results" section, the S1 table is indicated to contain the list of RePh genomes and the other group of 215 genomes. However, the S1 table does not contain data that indicate which accessions belong to the RePh group. The list of RePh accessions is available from fig S3B or from table S4 with slight effort, so I suggest to also include this information in table S1. Also, table S1 contains 238 accessions, whereas the text mentions 239 genomes.

Response: Table S1 was modified to include this information.

S2 table. The S2 table contains 119567 entries. This number is not consistent with the 107543 proteins mentioned page 5 for the number of full-length proteins. Even if we remove the 13007 proteins annotated as partial and listed in table S3, we do not obtain the number of proteins mentioned in the text. This table also contain redundant entries (106683 unique IDs).

Response: Yes, S2 Table has 119567 entries. The 107543 proteins mentioned in the text represent the number of full-length proteins (In total 12024 partial proteins were removed, including 11942 of unique proteins and 82 repeated ones). See also response to the first comment of the reviewer.

Still in table S2, a discrepancy is also observed for the number of PhAMEs: 7162 according to the text, and 7747 according to table S2.

Response: Out of 9981 predicted proteins with a predicted signal peptide, 663 are likely to be partial sequences and 2156 are likely to have additional predicted trans-membrane domains. 9981 – (663 + 2156) = 7162 PhAMEs. We don’t understand how the reviewer comes to 7742 entries.

Another problem with table S2 is that some proteins are associated with some RefSeq (GCF) GenBank assemblies, whereas according to table S1 the full genome set contains only GCA assemblies. Tables S1 and S2 should be consistent with each other and with other figures (S3B for instance for RePh assemblies).

Response: Table S1 and S3B were corrected to be consistent with S2 table. The genome assembly information used to calculate CheckM is now included in S2 table.

* S4 Table. Table S4 contains 7162 PhAMEs entries, as mentioned in the text. However, 2 of its entries (WP_011412484.1 and WP_041639883.1) appear in table S2 with no analysis by TMHMM or no prediction of signal peptide, which is contradictory to the definition of PhAMEs. Moreover, as already mentioned, table S4 contains 252 duplicated entries.

Response: Entry WP_011412484.1 in table 4 should read WP_011412648.1. We have corrected this.

WP_041639883.1 is the RefSeq annotation and this corresponds to ABC65597.1 in the NCBI annotation. Both correspond to AYWB locus tag 480. However, they have different predicted start codons. ABC65597.1 has the signal peptide and was also described in Bai et al., 2006 and Bai et al., 2009. Hence, we used the ABC65597.1 annotation and we added this NCBI number to table S2.

In addition to ABC65597.1, we noticed similar situations for NCBI entries ABC65803.1 and ABC65342.1. We included these entries in table S2.

We made adjustments elsewhere in the manuscript as appropriate.

* Figure S6 – legend – (A) SAP49 is a member of cluster C28 according to text and figure, not C41 as mentioned in legend.

Response: SAP49 belongs to C28- this has been corrected.

* Figure S7D –legend- subcluster cC42 is mentioned, whereas it should read "sC42".

Response: Typo corrected

* Figure S8A – On figure S8A, cluster C41 is associated with 8 rows/columns, and C55 with 7 rows/columns. However, according to table S4, cluster C41 contains 9 entries with structure predictions, and cluster C28 contains 8 entries. I have not checked the other clusters of fig S8A, but I wondered why not all of available structure predictions were represented on S8A fig? Also I suggest that authors indicate in the legend the meaning of "PPSP".

Response: S8A Fig is based on the unique (non-replicated) entries and did not include ‘duplicates’. See response to the first comment of the reviewer. To be consistent, we also removed the ‘duplicates’ for the entries under C80 and C185. We also changed ‘PPSP’ to ‘PhAMEs’ in the figure.

* Fig S14C It seems that "Alifatic" is usually written as "Aliphatic"

Response: Typo corrected

* Fig S15C (legend) "preciously" -> "previously"

Response: Typo corrected

* Fig S15D (legend) "In (E) one monomer is shown…"-> in (D)

Response: Typo corrected

* Fig S15E legend "TCP domain" in blue is incomplete

Response: Figure corrected

* Fig S16D "Lipophylic" shoud be written "Lipophilic"

Response: Typo corrected
